# Conditional Loss of BAF (mSWI/SNF) Scaffolding Subunits Affects Specification and Proliferation of Oligodendrocyte Precursors in Developing Mouse Forebrain

**DOI:** 10.3389/fcell.2021.619538

**Published:** 2021-07-15

**Authors:** Eman Abbas, Mohamed A. Hassan, Godwin Sokpor, Kamila Kiszka, Linh Pham, Cemil Kerimoglu, Andre Fischer, Huu Phuc Nguyen, Jochen F. Staiger, Tran Tuoc

**Affiliations:** ^1^Institute for Neuroanatomy, University Medical Center, Georg-August-University Göttingen, Göttingen, Germany; ^2^Department of Zoology, Faculty of Science, Alexandria University, Alexandria, Egypt; ^3^Department of Neuro- and Sensory Physiology, University Medical Center Göttingen, Göttingen, Germany; ^4^Protein Research Department, Genetic Engineering and Biotechnology Research Institute (GEBRI), City of Scientific Research and Technological Applications (SRTA-City), New Borg El-Arab City, Egypt; ^5^Department of Human Genetics, Ruhr University of Bochum, Bochum, Germany; ^6^German Center for Neurodegenerative Diseases, Göttingen, Germany; ^7^Cluster of Excellence “Multiscale Bioimaging: From Molecular Machines to Networks of Excitable Cells” (MBExC), University of Göttingen, Göttingen, Germany; ^8^Department for Psychiatry and Psychotherapy, University Medical Center Göttingen, Göttingen, Germany

**Keywords:** oligodendrocyte development, oligodendrogenesis, BAF complex, BAF155 and BAF170, OPC specification and proliferation

## Abstract

Oligodendrocytes are responsible for axon myelination in the brain and spinal cord. Generation of oligodendrocytes entails highly regulated multistage neurodevelopmental events, including proliferation, differentiation and maturation. The chromatin remodeling BAF (mSWI/SNF) complex is a notable regulator of neural development. In our previous studies, we determined the indispensability of the BAF complex scaffolding subunits BAF155 and BAF170 for neurogenesis, whereas their role in gliogenesis is unknown. Here, we show that the expression of BAF155 and BAF170 is essential for the genesis of oligodendrocytes during brain development. We report that the ablation of BAF155 and BAF170 in the dorsal telencephalic (dTel) neural progenitors or in oligodendrocyte-producing progenitors in the ventral telencephalon (vTel) in double-conditional knockout (dcKO) mouse mutants, perturbed the process of oligodendrogenesis. Molecular marker and cell cycle analyses revealed impairment of oligodendrocyte precursor specification and proliferation, as well as overt depletion of oligodendrocytes pool in dcKO mutants. Our findings unveil a central role of BAF155 and BAF170 in oligodendrogenesis, and thus substantiate the involvement of the BAF complex in the production of oligodendrocytes in the forebrain.

## Introduction

During mammalian brain development, the telencephalic radial glial cells (RGCs) generate diverse neuronal and non-neuronal cell types; the latter includes astrocytes, and oligodendrocytes (OLs) (Rowitch and Kriegstein, 2010). Among the glial cells, OLs are the specialized myelin-producing cells required for accelerated electrical impulse transmission in the central nervous system (CNS) (Richardson et al., 2006; Peru et al., 2008; Rowitch and Kriegstein, 2010; Nave and Werner, 2014; Sock and Wegner, 2019).

Generation of mature myelinating OLs is a sequential multistep process commencing with the specification of multipotent RGCs into oligodendrocyte precursor cells (OPCs) (Richardson et al., 2006; Rowitch and Kriegstein, 2010). Further in the oligodendroglial lineage progression, the highly proliferative and migratory OPCs acquire differentiative fate to become immature OLs, which subsequently undergo maturation to become myelinating OLs (Richardson et al., 2006; Rowitch and Kriegstein, 2010). Cell-tracing studies in the forebrain revealed that OPCs are generated from the dorsal telencephalon (dTel) and ventral telencephalon (vTel) RGCs. The first cohort of OPCs is generated at E12.5 from Nkx2.1-expressing progenitors in the medial ganglionic eminence (MGE), followed by the next wave of OPC generation at E15.5 from Gsx2-expressing progenitors in the lateral and caudal ganglionic eminences (LGE and CGE). The latest group of newly born OPCs are derived from Emx1-expressing progenitors in the neocortex at birth, and constitute the OPC pool in adulthood (Kessaris et al., 2006; Richardson et al., 2006; Naruse et al., 2017).

The progression of the OL lineage during brain development is under the control of key molecular regulation. The regulatory landscape of oligodendroglia is also characterized by enrichment with transcriptional and epigenetic factors, which are known to cooperate to bring about oligodendrogenesis (Emery and Lu, 2015; Koreman et al., 2018; Sock and Wegner, 2019). Notably, several studies have shown that chromatin regulators play important roles in the genesis and regeneration of OL in the developing vertebrate CNS (Marie et al., 2018; Elsesser et al., 2019; Parras et al., 2020).

In this study, we found that the conditional deletion of the ATP-dependent chromatin remodeling BRG1/BRM-associated factor (BAF) complex scaffolding subunits BAF155 and BAF170 in the developing forebrain resulted in improper specification and proliferation of OPCs leading to impairment of oligodendrogenesis. The observed defective oligodendrogenesis may have implication for abnormal myelination in the BAF155 and BAF170-deficient brain. In all, we provide evidence which consolidates the essential role of these two scaffolding BAF subunits in brain development by describing their involvement in the production of OLs in the CNS.

## Results

### Deletion of BAF155 and BAF170 in dTel Progenitors Perturb Oligodendrogenesis in the Postnatal Neocortex

We found in our previous investigations that inactivation of BAF complex by ablation of its scaffolding subunits BAF155 and BAF170 during early or late corticogenesis impairs the cortical progenitor pool, with concomitant disturbance of neurogenesis in the prenatal and early postnatal mouse cortex (Narayanan et al., 2015; Nguyen et al., 2018). Since neurons and glial cells are generated from a common RGC population (Zhuo et al., 2001; Gorski et al., 2002; Rowitch and Kriegstein, 2010), we predicted the likelihood of abnormal generation of glial cells in the absence of BAF155 and BAF170.

**FIGURE 1:**
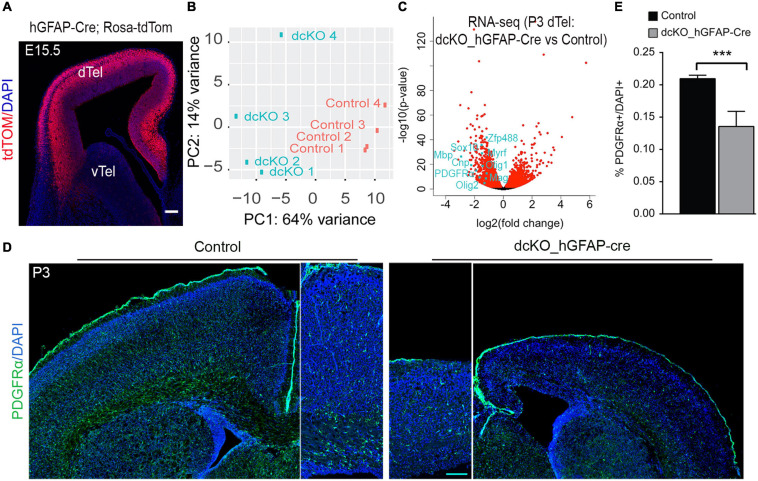
Cortex-specific loss of BAF155 and BAF170 led to decreased number of OPCs in postnatal cortex. **(A)** Coronal brain sections of hGFAP-Cre, Rosa-tdTom transgenic mouse embryo revealing the cortex-specific hGFAP-Cre recombinase activity pattern at E15.5 (Nguyen et al., 2018). **(B)** Graph showing the variance distribution or clustering in a principal component (PC) analysis of the four control and four mutants P3 hGFAP-Cre dcKO cortices used for the RNA sequencing. In a principal analysis **(C)** Volcano plot showing the distribution of genes upregulated and downregulate in the P3 hGFAP-Cre dcKO cortices compared with control. Examples of key oligodendrogenesis-related genes downregulate in the BAF155 and BAF170 mutant cortex are indicated. **(D)** Immunostaining of PDGFRα (green) in coronal sections of control and dcKO_hGFAP-Cre brains at P3. **(E)** Quantitative analysis comparing the percentage of the PDGFRα^+^ cells per total cortical cells (DAPI^+^) in the control and hGFAP-Cre dcKO cortex at P3. Data are presented as means ± SEMs; ***p < 0.001; Experimental replicates (*n*) = 7. dTel, dorsal telencephalon; vTel, ventral telencephalon. Scale bars = 200 μm **(A**,**D)**.

To ascertain this possibility, we examined the transcriptome of the mouse cortex at P3 following hGFAP-Cre-mediated deletion of BAF155 and BAF170 in the neocortex (Supplementary Table 1). In the hGFAP-Cre transgenic mice, the recombinase activity is reported to be prominently active in dTel RGCs by E15.5 (Figure 1A; Zhuo et al., 2001; Nguyen et al., 2018). In dcKO_hGFAP cortex at P3, there were 3199 down- and 2804 up-regulated genes as compared with control cortex (Figures 1B,C and Supplementary Table 1, *p* < 0.05). A closer look at the differentially expressed genes revealed decrease in the expression key genes (e.g., Olig2, Olig1, PDGFRα, Mbp, Mag, Sox10, Myrf, ZFP488, CNP) involved in oligodendroglial development in the P3 mutant (dcKO_hGFAP-Cre) cortex as compared with control (Figure 1C). Indeed, histological investigation showed that PDGFRα-immunoreactive oligodendrocyte precursor cells and nascent oligodendrocytes, are demonstrably lost in the dysgenic P3 dcKO_hGFAP-Cre neocortex as compared with control (Figures 1D,E).

Together, our transcriptomic analysis and initial *in vivo* examination of the early postnatal developing neocortex lacking BAF155 and BAF170 point to a plausible involvement of these scaffolding subunits of the BAF complex in the generation of oligodendrocytes.

### BAF Complex Scaffolding Subunits BAF155 and BAF170 Are Highly Expressed in Oligodendrocyte Lineage

**FIGURE 2:**
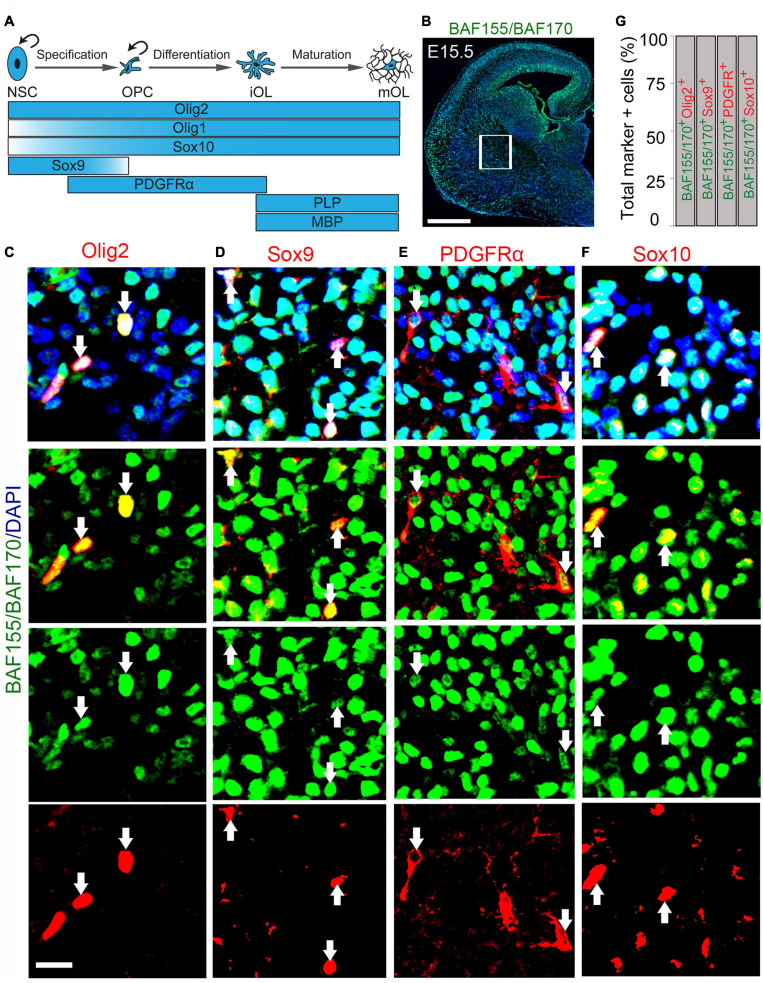
BAF155 and BAF170 expression in the oligodendroglial lineage in the ventral telencephalon. **(A)** Diagram illustrating the stepwise oligodendrocyte lineage progression and the expression pattern of key molecular drivers. Curved arrows denote proliferation. **(B–F)** Micrographs showing BAF155 and BAF170 expression **(B)** alongside the oligodendrocyte lineage markers Olig2, Sox10 for pan-oligodendrocyte **(C,F)**, Sox9 for glioblasts **(D)**, and PDGFRα for OPCs **(E)** in coronal sections of the mouse forebrain at E15.5 using immunohistochemistry. **(G)** Bar graph showing quantitative estimation of proportion of Olig2, Sox9, PDGFRα, and Sox10 positive cells with BAF155 and BAF170 expression. Inserted box shows selected area for quantification. Arrows refer to the oligodendrocytic lineage expressing BAF155 and BAF170. Experimental replicates (*n*) = 3. NSC, neural stem cell; OPC, oligodendrocyte precursor cell; iOL, immature oligodendrocyte, mOL, mature oligodendrocyte. Scale bars = 200 μm **(B)**, 25 μm **(C)**, 50 μm **(D–F)**.

Given the significant reduction in the number of OPCs consequent to BAF155 and BAF150 abolishment in the late developing neocortical neuroepithelium, we decided to examine the expression of BAF155 and BAF150 in OL lineage (Figure 2A). Such expression analysis was performed in the striatum, a developing forebrain region in the vicinity of the hub of oligodendrocyte precursor generation (i.e., the ganglionic eminences) found in the vTel (Figure 2B). Immunostaining of the E15.5 mouse cortex revealed observable colocalization of key OL lineage markers (Olig2, Sox9, PDGFRα, and Sox10; Figure 2A) with BAF155 and BAF170 (Figures 2B–F). Quantitative analysis of the co-expression of BAF155/BAF170 with Olig2, which labels all the cells of the OL lineage (Figure 2A; Silbereis et al., 2014), showed that all of the Olig2^+^ cells prominently express BAF155 and BAF170 in the presumptive striatum (Figures 2C,G, arrows). This observation of BAF155 and BAF170 expression in Olig2 cell lines was supported by similar analysis with the previously mentioned additional OL lineage markers (Sox9, PDGFRα, and Sox10). The further quantitative analysis indicated that all of Sox9^+^ glial cells, PDFGRα^+^ OPCs, and Sox10^+^ OLs display expression of BAF155 and BAF170 (Figures 2D–G). Replication of this expression analyses in the E18.5 striatum reflected consistency with the expression profile observed in the E15.5 brain (data not shown).

Put together, our results reveal enrichment of BAF155 and BAF170 expression in the majority of cells that constitute the OL lineage. The findings suggest a possible involvement of these core BAF complex subunits in oligodendrogenesis.

### Ablation of BAF155 and BAF170 in Early Oligodendrocyte Precursors Caused Depletion of Oligodendrocyte Lineage Population

In order to clearly determine the effect of loss of BAF155 and BAF170 on oligodendrogenesis in the forebrain, we specifically deleted BAF155 and BAF170 in early OL precursors. To achieve the knockout of BAF155 and BAF170 in early OL precursors, we crossed mice carrying floxed alleles for BAF155 (Choi et al., 2012) and BAF170 (Tuoc et al., 2013) with the OL-targeting Cre line Olig2 (Dessaud et al., 2007), to generate dcKO_Olig2-Cre mutants. Unlike the recombinase activity of hGFAP-Cre, which was found in the majority of dTel cells (Figure 1A), that of Olig2-Cre was observed in MGE (already at E12.5) and in LGE/CGE (from onward E15.5) in the vTel (Supplementary Figure 1A).

**FIGURE 3:**
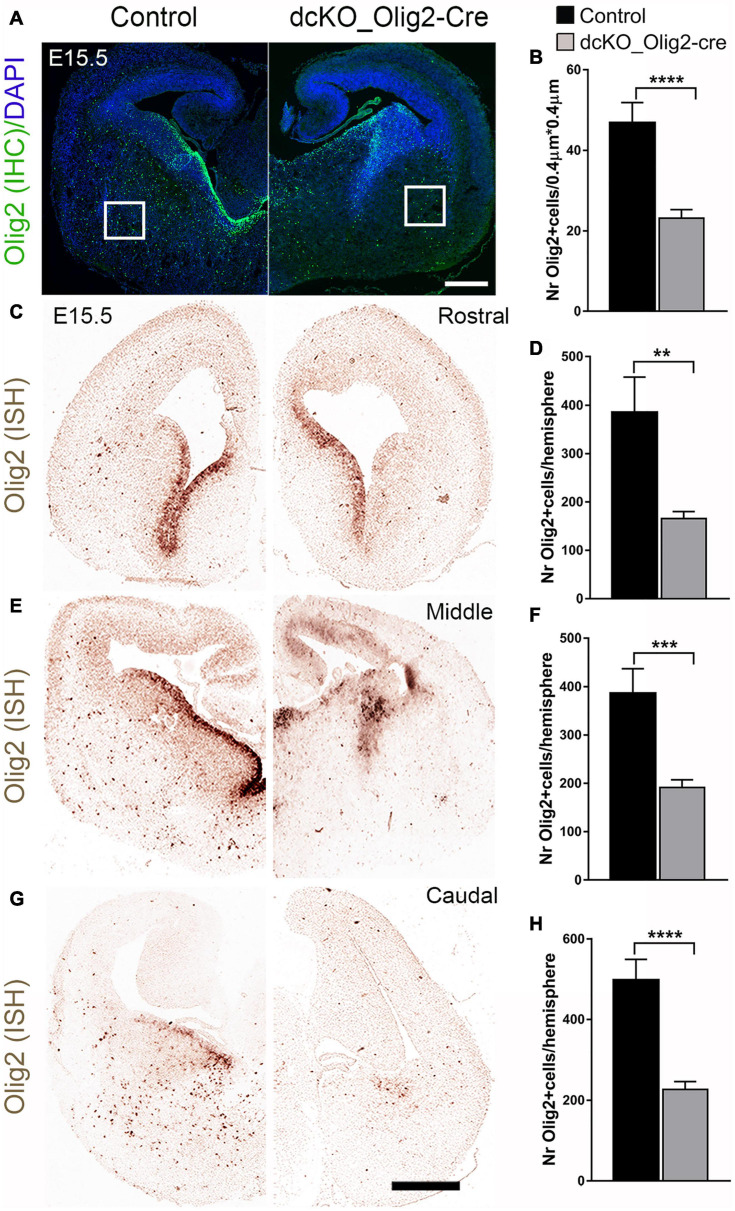
Decrease in the number of Olig2-expressing cells due to loss of BAF155 and BAF170 in ventral telencephalon. **(A,B)** Immunohistochmical micrograph **(A)** and quantitative analyses **(B)** indicating diminished number of Olig2^+^ cells caused by loss of BAF155 and BAF170. **(C,E,G)** *In situ* hybridization micrographs using Digoxigenin-labeled Olig2 RNA probes in the control and dcKO_Olig2-Cre E15.5 mouse telencephalon along the rostral–caudal axis. **(D,F,H)** Statistical analyses indicate significant depletion of Olig2^+^ cells in the rostral middle, and caudal levels of the dcKO_Olig2-Cre mutant telencephalon compared with control. Data are expressed as means ± SEMs. Experimental replicates (*n*) = 6; **p ≤ 0.01, ***p ≤ 0.001, ****p ≤ 0.0001. Scale bars = 200 μm **(A)**, 100 μm **(D)**.

To validate our dcKO_Olig2-Cre model, we compared expression of BAF155/BAF170 in controls and mutants at E12.5 in the MGE VZ, where Olig2-Cre activity is high in mutants (Supplementary Figure 1B). Indeed, expression of these subunits was completely lost in MGE VZ at E12.5 (Supplementary Figure 1B). Furthermore, our analysis also revealed that expression of BAF155/BAF170 was eliminated in Olig2^+^/PDGFRα^+^ OPCs in the mutant striatum at E15.5 (Supplementary Figure 1C). Upon immunohistological examination of the E15.5 dcKO_Olig2-Cre forebrain, we noticed severe disturbance of oligodendrogenesis following inactivation of BAF155 and BAF170 in OL lineage. Quantitative assessment revealed a significant reduction in the population of cells in the early embryonic (E15.5) dcKO_Olig2-Cre striatum immunopositive for the pan OL marker Olig2 compared with control (Figures 3A,B). Results of *in situ* hybridization of *Olig2* transcripts consistently showed a reduction in Olig2^+^ cells in the striatum from the rostral to caudal aspects of the E15.5 dcKO_Olig2-Cre mutant forebrain compared with control (Figures 3C–H). The loss of Olig2 expression was dramatically severe in the whole extent of the late embryonic (E18.5) vTel when mutant littermates were compared with control (Supplementary Figure 2).

Additional investigation of the dcKO_Olig2-Cre forebrain was performed using forebrain tissue from E15.5 embryos, and the riboprobes for *Olig1* and *Sox10*, which can be used as an alternative marker for the OL lineage (Wegner, 2001). In consonance with the Olig2 analysis, we found overt decrease in the cells expressing *Olig1* and *Sox10* transcripts (Supplementary Figures 3–5). Again, as observed for *Olig2* expression, the decrease in the number of Olig1^+^/Sox10^+^ cell lineage was identified in the rostral, middle, and caudal aspects of the mutant forebrain compared with control (Supplementary Figures 3–5).

The results presented here, provide evidence that the function of the scaffolding BAF complex subunits BAF155 and BAF170 are necessary for the proper establishment of the OL lineage in the developing forebrain.

### BAF155 and BAF170-Deficient vTel Displays Defective Specification and Proliferation of Oligodendrocyte Precursors

The BAF complex is essential for proper lineage progression (differentiation) of OPCs to nascent (immature) OLs and further differentiation to mature myelinating OLs (Yu et al., 2013; Bischof et al., 2015). Whether its subunits are also required for earlier stages of OL development is largely unknown.

**FIGURE 4:**
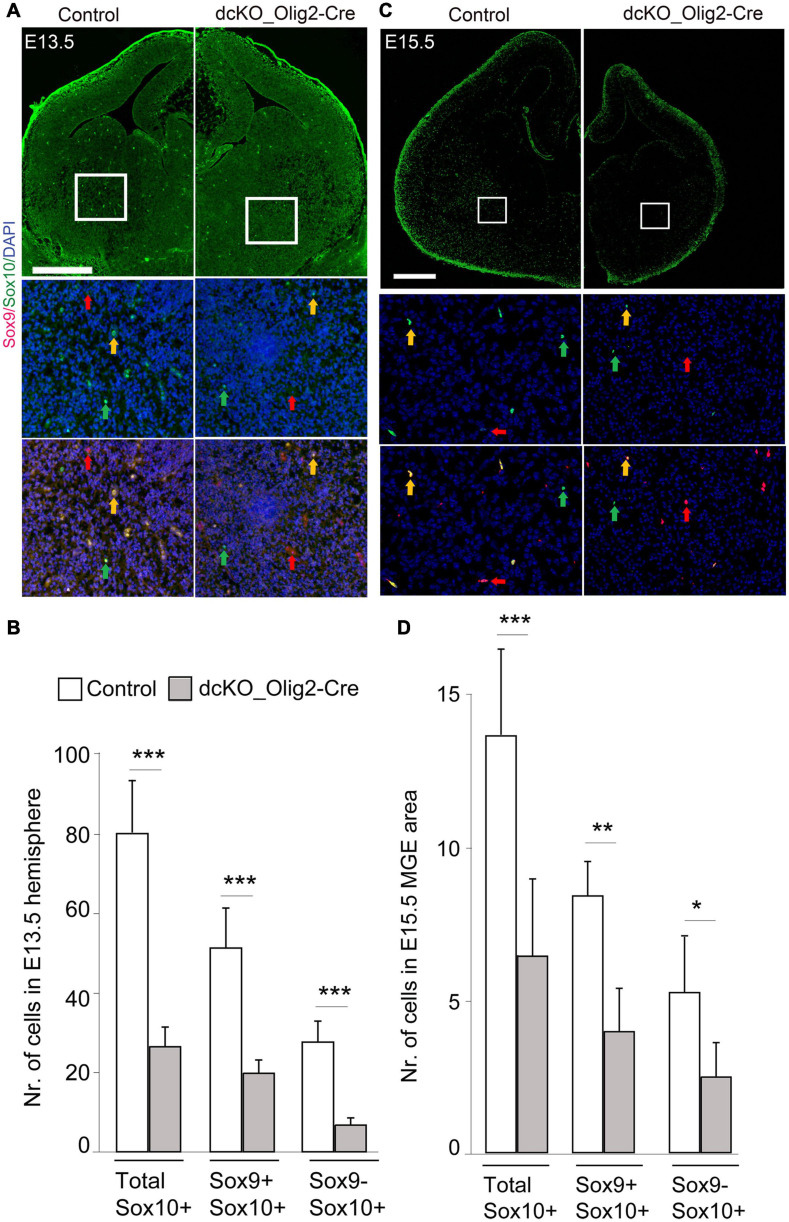
Specification of oligodendrocyte precursors is impaired in the dcKO_Olig2-Cre. **(A,C)** Immunomicrographs showing Sox9 and Sox10 in the E13.5 **(A)** E15.5 **(C)** control and dcKO_Olig2-Cre forebrain. Selected regions of the striatum are shown at higher magnification. Yellow arrows point to cells co-expressing Sox9 and Sox10. Green arrows point to cells expression only Sox10. Red arrows point to cells expressing only Sox9. DAPI counter staining is shown. **(B,D)** Bar plots showing statistical difference in the number of cells (co)expressing Sox9, Sox10, or Sox9/Sox10 in the E13.5 **(B)** E15.5 **(D)** control and dcKO_Olig2-Cre forebrain. Data are shown as means ± SEMs. Experimental replicates (*n*) = 6; *p ≤ 0.01, **p ≤ 0.001, ***p ≤ 0.0001. Scale bars = 100 μm.

In the CNS, TF Sox9 is strongly expressed first in NSCs, and later in glioblasts and astrocyte precursors, thus making it essential for proper development of both oligodendrocytes and astrocytes (Stolt et al., 2003). During oligodendroglial development, Sox10 expression starts earlier than the expression of OPC markers such as PDFGRα and is maintained until mature OL stage (Wegner, 2001; Stolt et al., 2003). To examine whether dual removal of BAF155/BAF170 causes defective specification of OPCs, we examined the co-expression of Sox9 and Sox10, which label OPCs derived from NSC during the specification stage of oligodendrocyte lineage progression (Figures 2A, 4). In control striatum at E13.5 and E15.5, many Sox10^+^ cells are also immunoreactive with Sox9 (Figures 4A,C, yellow arrows). Remarkably, the number of both Sox10^+^/Sox9^+^ cells (yellow arrows) and Sox10^+^/Sox9^–^ OLs (green arrows) are decreased in the dcKO mutants (Figures 4A–D). This suggests that expression of BAF155 and BAF170 is necessary for the proper specification of the OL lineage in the developing forebrain.

In addition to the OL specification phenotype, we investigated whether the loss of BAF155/BAF170 causes defective proliferation of OPCs in the dcKO mutants. During oligodendrogenesis, committed OPCs divide either symmetrically to produce a pair of cycling OPCs or asymmetrically to give rise to one proliferative OPC and one differentiated OL (Rowitch and Kriegstein, 2010).

**FIGURE 5:**
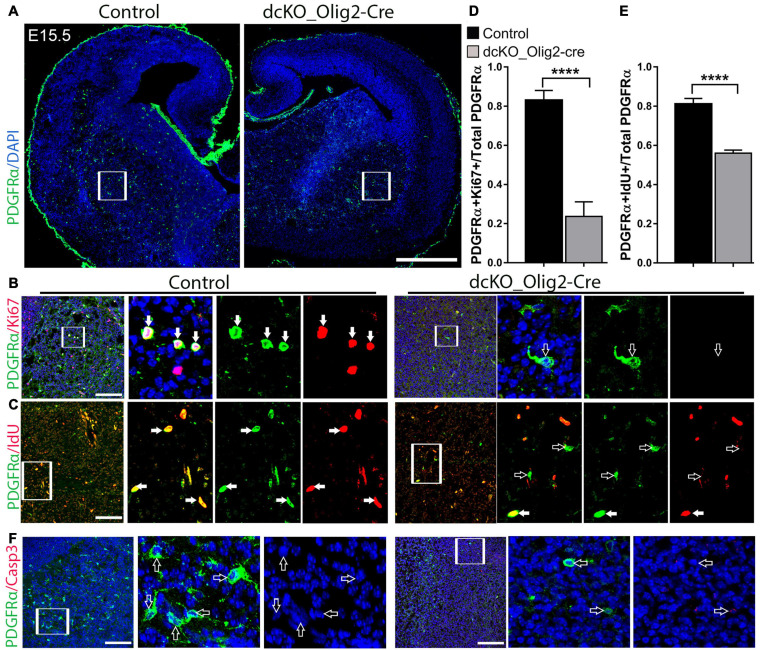
Conditional removal of BAF155 and BAF170 leads to an impaired proliferative capacity of OPCs. **(A)** Images showing immunostaining of PDGFRα-labeled oligodendrocyte precursor cells in the dcKO E15.5 forebrains compared with controls. **(B,C)** High magnification images of the outlined regions (inserted boxes) of the ventral telencephalon showing immunolabeling of PDGFRα and Ki67 **(B)** and PDGFRα and IdU **(C)** to mark proliferating oligodendrocyte precursor cells in the E15.5 control and mutant forebrain. Filled arrows show PDGFRα positive oligodendrocyte precursor cells expressing Ki67 or IdU, and empty arrows point to PDGFRα oligodendrocyte precursor cells negative for Ki67 or IdU. **(D,E)** Bar charts showing significant reduction of the ratio of PDGFRα^+^ and Ki67^+^ per total PDGFRα^+^ cells as well as the ratio PDGFRα^+^ and IdU^+^ per total PDGFR^+^ cells in the dcKO_Olig2-Cre ventral telencephalon compared with control. **(F)** High magnification images showing PDGFRα, Sox10 (violet) and Casp3 immunostaining in the E15.5 ventral telencephalon. Empty arrows point to PDGFRα^+^ and Sox10^+^ cells lacking Casp3 expression. Data are presented as means ± SEMs; ****p ≤ 0.0001; Experimental replicates (*n*) = 6. Scale bars = 200 μm **(A)**, 50 μm **(B,C,F)**.

We previously found that inactivation of the BAF complex via deletion of BAF155 and BAF170 in neural stem cells leads to defective cell cycle dynamics in neural precursors (Narayanan et al., 2015; Bachmann et al., 2016; Nguyen et al., 2018). On such basis, and the observed aberrant reduction in the oligodendrocyte population in the dcKO_Olig2-Cre forebrain, we sought to find out the effect of BAF155 and BAF170 on the proliferative capacity of OPCs in the developing brain. To do this, we performed double immunostaining using antibodies against PDGFRα to mark the OPCs, and Ki67 to indicate OPCs undergoing active proliferation in the E15.5 mutant and control brain (Figures 5A,B). Whereas the vast majority of OPCs in the examined control striatum were found with PDGFRα and Ki67 co-labeling (Figures 4B,D, filled arrows), the PDGFRα^+^ OPCs in the dcKO_Olig2-Cre mutant striatum displayed massive lack of Ki67 labeling (Figures 5B,D, empty arrows). Additional evidence indicating altered proliferation fate in OPCs due to lack of BAF155 and BAF170 was obtained from experiment in which pulse labeling using the Thymidine analog IdU, which incorporates into the DNA of cell at S phase of the cell cycle, showed very few PDGFRα-expressing OPCs positive for IdU in the mutant striatum compared with control (Figures 5C,E, filled arrows).

Since most of the mutant OPCs are abnormally non-proliferative and depleted in number, we were curious to find out whether apoptosis plays a role in the observed phenotype. Our investigation of apoptotic activity using immunohistochemical staining of Casp3, a protein maker for apoptosis, revealed comparable Casp3 staining in the E15.5 striatum in the control and dcKO_Olig2-Cre forebrain (Supplementary Figures 5F,G).

Together, this part of our investigations shows that in addition to defective specification, BAF155 and BAF170-deficient OPCs are less proliferative, thus implying their essentiality in keeping OPCs in the cell cycle and ensuring renewal and/or maintenance of the OL progenitor pool. As a consequence, the pool of PDGFRα^+^ OPCs is virtually depleted in the dcKO_Olig2-Cre forebrain compared with control (Figure 5A).

### Number of PLP^+^, MBP^+^ Oligodendrocytes Is Reduced in BAF155 and BAF170 Mutant Forebrain at E18.5

**FIGURE 6:**
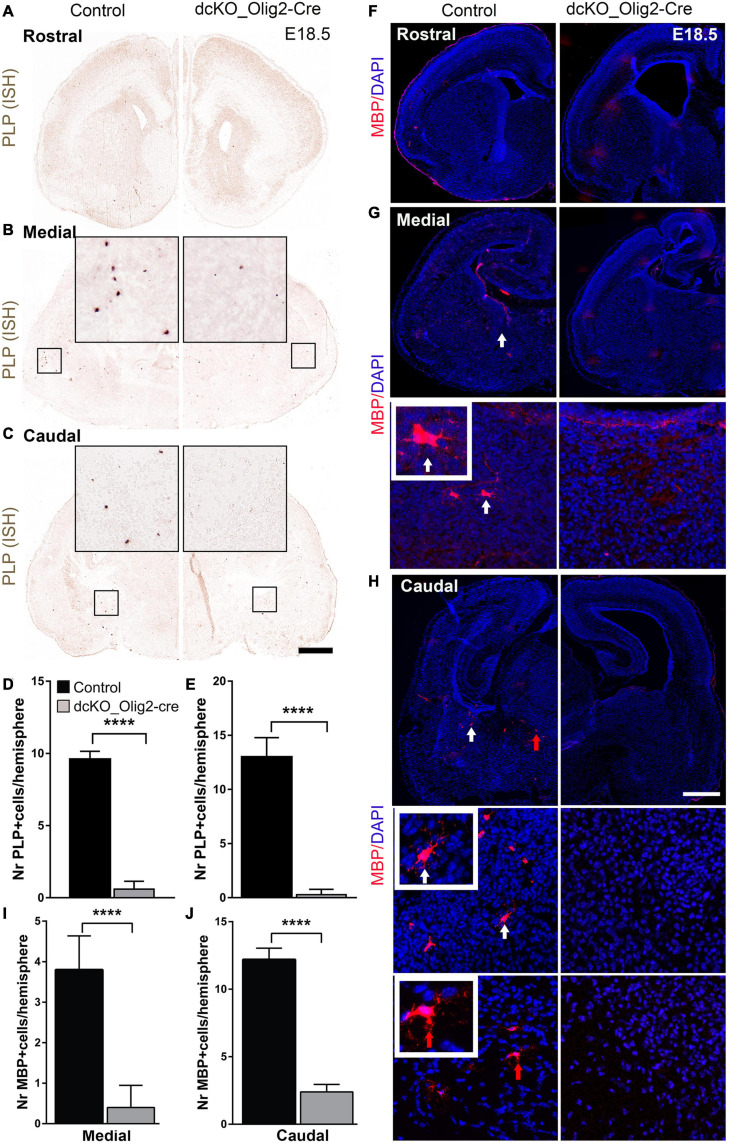
Number of PLP^+^, MBP^+^ oligodendrocytes is reduced in dcKO_Olig2-Cre mutant forebrain at E18.5. **(A–C)** *In situ* hybridization images showing the E18.5 control and dcKO_Olig2-Cre forebrain coronal sections with riboprobed for PLP expression. **(D,E)** Bar charts showing quantification of PLP-expressing cells in the E18.5 control and dcKO_Olig2-Cre at the middle **(D)**, and caudal **(E)** levels of the ventral telencephalon Experimental replicates (*n*) = 5, Scale bars = 100 μm. **(F–H)** Micrographs showing the rostral, middle, and caudal sections of the E18.5 mouse forebrain immunostained with MBP and with DAPI counterstaining. Inserted images are higher magnification of MBP-expressing cells indicate by white and red solid arrows. **(I,J)** Bar graph showing significantly diminished number of cells expression MBP in the E18.5 control striatum compared with that of dcKO_Olig2-Cre. Note that PLP^+^, MBP^+^ cells are very rare in E18.5 control and dcKO_Olig2-Cre forebrain at rostral level and are not included in statistical analysis. Data are presented as means ± SEMs; ****p ≤ 0.0001); Experimental replicates (*n*) = 6. Scale bars = 100 μm.

Because the defective specification, proliferation, and diminished pool of OPCs in the dcKO_Olig2-Cre forebrain may have implication for OL generation, we searched for evidence indicating altered pool of differentiated OLs in the absence of BAF155 and BAF170. To that end, we carried out *in situ* hybridization to visualize the expression of the proteolipid protein (PLP/DM-20) transcript known to identify mature OL (Figure 2A). Interestingly, the PLP signal in the E18.5 dcKO_Olig2-Cre forebrain is reduced compared with control (Figures 6A–E). As such, whereas cells brightly labeled with PLP probe can be found in the striatum of control forebrain, the dcKO_Olig2-Cre forebrain demonstrably lacked PLP staining at comparable brain section levels (Figures 6A–E). Consistent with ISH analysis with PLP probe, IHC analysis with MBP antibody revealed a few MBP^+^ mOLs in the middle and caudal sections of control vTel at E18.5 (Figures 6F–H). Some of MBP^+^ mOLs display their branching process (Figures 6G,H, in close-up). As expected, population of MBP^+^ mOLs is diminished in dcKO mutants (Figures 6F–J).

**FIGURE 7:**
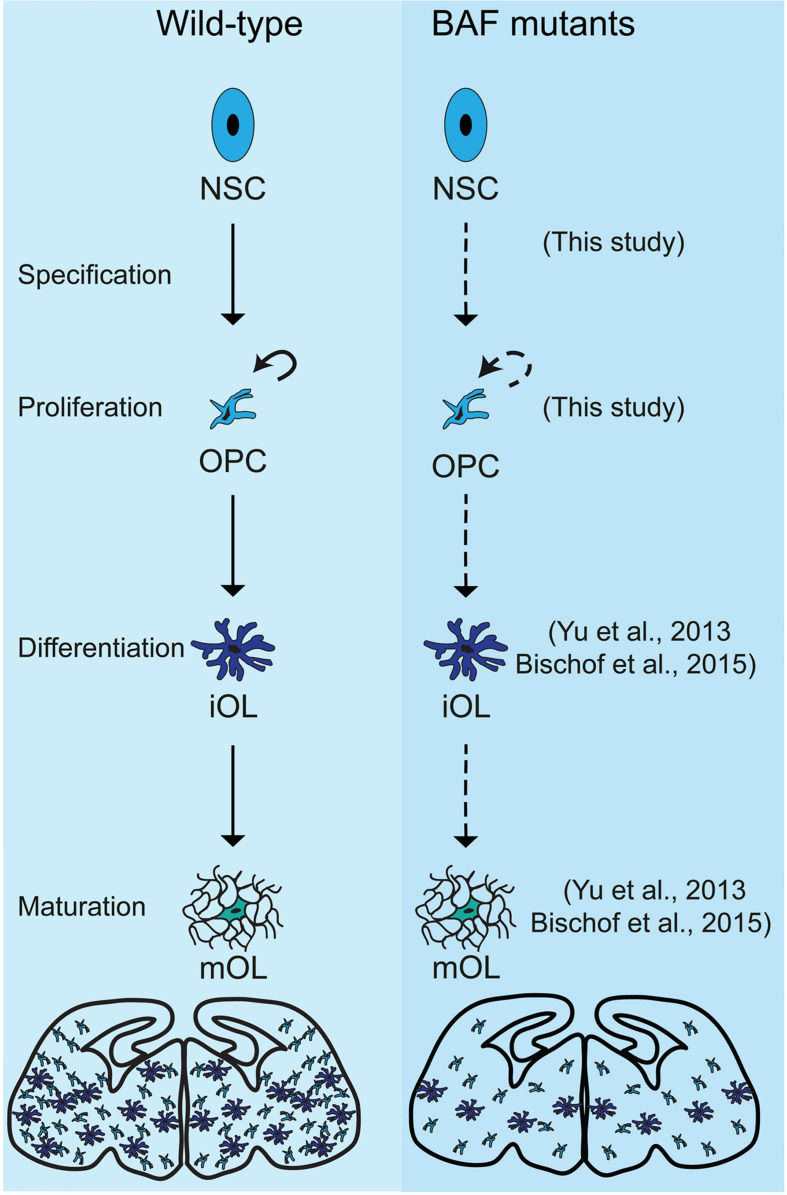
Schema summarizing the effects of loss of chromatin remodeling BAF complex activity on oligodendroglial lineage progression in BAF155/BAF170dcKO and Brg1cKO mutants. The illustration shows a comparison of the progression of oligodendrocyte development in the developing mouse forebrain between control (Wild-type, in the left panel) and BAF mutants (in the right panel), i.e., BAF155/BAF170cKO mutants (this study) and Brg1cKO mutants (Yu et al., 2013; Bischof et al., 2015). In control, multipotent neural stem cell (NSC) gives rise to oligodendrocyte precursor cells, which are able to proliferate (shown by curved arrow) and differentiate into immature oligodendrocyte (imOL). The imOL undergoes maturation to become fully myelinating mature oligodendrocyte (mOL). However, in the absence of BAF155 and BAF170, the OPC is improperly specified from the mutant NSC and undergoes reduced proliferation (this study). In addition, in the absence of Brg1, OPCs were unable to differentiate and mature properly (Yu et al., 2013; Bischof et al., 2015). The diagrams at the end of the schema depict the overall depletion of OLs in the BAF mutant brain compared with control. References to key studies that reported phenotypes due to ablation of the BAF complex are indicated. Dashed-arrows are used to indicate defective developmental processes.

Our previous study indicated that many BAF subunits are lost via proteasomal degradation in response to the ablation of the two scaffolding subunits BAF150/BAF170 in ESCs, in cortical cells (Narayanan et al., 2015) and in olfactory epithelium (Bachmann et al., 2016). To provide certain link between BAF155/BAF170 and OPC differentiation to OL, we examined the expression of Brg1 subunit, which was reported to be crucial for the lineage progression of OPCs (Yu et al., 2013; Bischof et al., 2015), in our dcKO_Olig2-Cre brain model. As expected, we found expression of Brg1 to be completely ablated in dcKO MGE, where Cre activity is highest (Supplementary Figure 6). Our data suggest a possible involvement of BAF155/BAF170 in OPC differentiation and OL maturation by direct control of the expression of Brg1. Altogether, our findings and that of others demonstrate that aside the proper lineage progression of OPCs to immature OLs which subsequently differentiate into mature OLs capable of myelination (Yu et al., 2013; Bischof et al., 2015), BAF complex is required for OPC specification and proliferation in the mouse forebrain (this study, Figure 7).

## Discussion

Emerging evidence point to the implication of chromatin regulation mechanisms in the control of oligodendroglial development and myelination in the nervous system (Lessard et al., 2007; Weider et al., 2012; Limpert et al., 2013; Ninkovic et al., 2013; Yu et al., 2013; Bischof et al., 2015; Emery and Lu, 2015; Alver et al., 2017; Marie et al., 2018). The mammalian BAF complex comprises heterogeneous subunits, with core subunits including BRG1/Smarca4 and BRM/Smarca2 functioning as ATPases, and BAF155 and BAF170 acting as scaffolding proteins. Through the use of energy obtained from the breakdown of ATP, the BAF complex is able to drive structural changes in chromatin leading to increase in the accessibility to gene-encoding regions, and thus influencing gene expression (reviewed in Hargreaves and Crabtree, 2011; Sokpor et al., 2018).

Although information is building up on the role of the BAF complexes in driving OL development (Matsumoto et al., 2006; Weider et al., 2012; Marathe et al., 2013; Yu et al., 2013; Bischof et al., 2015; He et al., 2016; Matsumoto et al., 2016; Zhao et al., 2018), major gaps exist in our in-depth understanding of how the chromatin remodeling BAF complex or its constituents feature in oligodendrogenesis. Our previous work have identified the phenomenal role of the BAF complex subunits BAF155 and/or BAF170 in orchestrating several aspects of neural development (neurogenesis), including neural progenitor proliferation and differentiation, and neuronal migration. As a result, BAF155 and BAF170 ablation leads to abnormal forebrain development (Narayanan et al., 2015, 2018; Bachmann et al., 2016; Nguyen et al., 2016, 2018; Sokpor et al., 2021). A question that remained was whether loss of BAF155 and BAF170 has implications for abnormal gliogenesis.

In the current study, our detail examination of Olig2-Cre activity in the transgenic reporter line (i.e., Olig2-Cre; Rosa-tdTom) revealed that recombinase activity is found in the entire MGE from E12.5 and in LGE and CGE from E15.5 (Supplementary Figure 1A). Thus, Olig2-Cre line is suitable for investigating the role of BAF155/BAF170 in the first (at MGE) and second wave (at LGE and CGE) of oligodendrogenesis in the vTel. Similarly, hGFAP-Cre is extensively active in majority of cortical cells from E15.5 onward (Figure 1A). Thus, it is also appropriate for studying the third wave of OL generation in cortex. Altogether, Olig2-Cre and hGFAP-Cre lines are alternative genetic tools for investigation of all three developmental oligodendroglial waves.

We present evidence indicating that the BAF complex scaffolding subunits BAF155 and BAF170 are essential for specification and proliferation of OPCs and oligodendrogenesis in the developing mouse forebrain. Due to the indispensability of BAF155 and BAF170 in maintaining the integrity of the entire BAF complex (Narayanan et al., 2015), suggesting a possibility that dual loss of BAF155/BAF170 caused stronger defects in development than that by loss of individual BAF subunits. As evidenced in our RNA sequencing analysis, the gene expression program that supports oligodendrogenesis is abnormally downregulated due to loss of BAF155 and BAF170 in the early postnatal developing neocortex. The BAF155 and BAF170-dependent OL production phenotype was made clearer and more conclusive by specifically deleting BAF155 and BAF170 in the vTel neuroepithelium, from which OPCs are mainly generated in the embryonic brain (Kessaris et al., 2006; Richardson et al., 2006). Indeed, histological examination of the developing mutant (dcKO) forebrain revealed striking perturbation in oligodendrogenesis caused by BAF155 and BAF170 silencing. Of note, the expression of BAF155 and BAF170 was found to be supportive for specification and proliferation of OPCs. Based on our investigations with Ki67 and IdU immunohistochemistry, it is not far-fetched to reason that OPCs lacking BAF155 and BAF170 may aberrantly adopt a quiescent fate, especially given that apoptosis does not account for their pool size depletion with reference to control (Figures 4, 5). Our finding thus uncovers a possible role for the chromatin remodeling BAF complex in OPC specification and proliferation via expression of its scaffolding subunits BAF155 and BAF170.

The ATPase subunit of the BAF complex Brg1 is essential for proper differentiation of OPCs into immature and then mature OLs (Yu et al., 2013; Bischof et al., 2015). In addition, the deletion of Brg1 before the onset of OPC specification by using the Brn4-Cre deleter caused very weak and delayed induction of Sox10 expression (Bischof et al., 2015). Because Sox10 is an essential regulator of PDGFRα expression (Finzsch et al., 2008), it implies that Brg1 is essential for the correct induction of early OPC markers such as Sox10 and PDGFRα. Nevertheless, whether this subunit is also required for earlier stages of OL development still needed further investigations. During the formation of the BAF complex, alternative ATPase core BRM (Ho and Crabtree, 2010; Hargreaves and Crabtree, 2011) may substitute for the loss-of-function of BRG1 in the BRG1 mutants. It therefore would also be interesting for future investigation to focus on determining if BRM can effectively substitute for BRG1 to allow the recruitment of the BAF complex to gene loci that drive oligodendrogenesis.

Notably, the developing mutant forebrain presented with demonstrable depletion of the population of Sox10^+^ cells. To link this finding to similar outcome caused by BRG1 ablation in brain (Yu et al., 2013; Bischof et al., 2015), it is likely that the double deletion of the scaffolding BAF complex subunits BAF155 and BAF170 resulted in the proteasomal degradation of BRG1 (Narayanan et al., 2015) leading to phenocopy of defective OPC differentiation. Moreover, and again, similar to phenotype of Brg1 cKO mutants (Yu et al., 2013; Bischof et al., 2015), we observed a reduced expression of PLP, which marks mature myelin-forming OLs in the entire developing mammalian forebrain. This suggests that the few iOLs formed in the dcKO brain are incapable of maturation and myelination. Therefore, our investigation suggests the plausible role of the chromatin remodeling factors BAF155 and BAF170 in orchestrating oligodendroglial differentiation and maturation in the developing brain by directly controlling the expression of Brg1 (Supplementary Figure 6). It should be noted however that the expression of Olig2 is found not only in the OL lineage, but also in other cell types such as RGCs, IPCs, and neurons (Li et al., 2021). Therefore, the loss of BAF155 and BAF170 in dcKO_Olig2-Cre mutants is not restricted to the OL lineage, but also occurs in other cell types. Thus, defective specification and proliferation of OPCs could be a result of cell autonomous and/or non-autonomous mechanisms. It is worth considering using a more OL-restricted Cre line such as Sox10-Cre, or PDGFRα-Cre to examine the above mechanisms. Additionally, it would be informative to investigate whether BAF155 and BAF170 mediate the recruitment of the BAF complex to enhancer elements required for OPC specification and proliferation.

The importance of the tight transcriptional, chromatin and epigenetic regulation of oligodendroglial development in the CNS is emphasized by the several critical factors identified to orchestrate the transformations of neural stem cells or OPCs to mature myelinating OLs (Rowitch, 2004; Wegner, 2008; Li et al., 2009; Emery, 2010; Lu and Barca, 2012; Galloway and Moore, 2016). Of emerging interest, chromatin and epigenetic regulators are extensively shown to participate in the development of oligodendrocytes, myelination, and myelin repair in the brain (Copray et al., 2009; Yu et al., 2013; Bischof et al., 2015; Emery and Lu, 2015; Matsumoto et al., 2016; Koreman et al., 2018; Egawa et al., 2019; Tiane et al., 2019). Therefore, ablation of such chromatin and epigenetic factors is expected to call forth structural and functional impairment in the developing or adult brain. Indeed, the impaired development of OL lineage caused by defective chromatin and epigenetic regulation of the various steps involved, is linked to pathophysiological changes leading to neurodevelopmental and neurodegenerative disorders (Maki et al., 2013; Ohtomo et al., 2018; Lu et al., 2019; Berry et al., 2020; Samudyata et al., 2020). Thus, it is plausible that the reported role of BAF155 and BAF170 in OL development (this study) partly highlights the white matter anomalies associated with documented syndromic and non-syndromic disorders associated with BAF complex dysfunction (Sokpor et al., 2017).

Altogether, our findings demonstrate the significance of BAF155 and BAF170 subunits of the BAF complex in driving the specification and proliferation of OPCs. Future study is required to determine the function of BAF155 and BAF170 in oligodendrocyte differentiation and eventual maturation to be able to carry out myelination in the CNS. Describing a comprehensive mechanistic role for the regulatory function of BAF155 and BAF170 would boost our knowledge of the importance of chromatin modulators in myelination-dependent brain morphogenesis, and possibly lend therapeutic ideas for averting neurologic disorders in the event of dysregulation.

## Materials and Methods

### Transgenic Mice

*BAF155^*fl*/f*l*^* (Choi et al., 2012), *BAF170^*fl*/f*l*^* (Tuoc et al., 2013), Olig2-Cre (Dessaud et al., 2007), and hGFAP-Cre (Zhuo et al., 2001) mice were maintained in a C57BL6/J background. Animals were handled in accordance with the German Animal Protection Law.

### Generation of *dcKO* Mutants

To ablate the function of *BAF155* and *BAF170* in dTel progenitors, we used the hGFAP-Cre line as a driver for recombinase activity (Zhuo et al., 2001). We crossed *BAF155^*fl/*+^; BAF170^*fl/*+^; hGFAP-Cre^*pos*/+^* with *BAF155^*fl/fl*^; BAF170^*fl/fl*^* mice to generate dcKO_hGFAP-Cre. Similarly, to inactivate the function of these BAF subunits in vTel progenitors and the Olig2^+^ cell lineage, we used the Olig2-Cre line (Dessaud et al., 2007), which was obtained from Till Marquardt lab (Müller et al., 2014). We crossed *BAF155^*fl/*+^; BAF170^*fl/*+^; Olig2-Cre^*pos/*+^* with *BAF155^*fl/fl*^; BAF170^*fl/fl*^* mice to generate dcKO_Olig2-Cre. Both dcKO_hGFAP-Cre and dcKO_Olig2-Cre mutants died soon after birth. The comparative expression analyses were performed on matched sections of the mutant (*BAF170^*fl/fl*^; BAF155^*fl/fl*^; Cre ^pos/+^*) vs. control brains (*BAF155^*fl/*+^; BAF170^*fl/*+^; Cre ^pos/+^*).

### Antibodies

The following polyclonal (pAb) and monoclonal (mAb) primary antibodies used in the study were obtained from the indicated commercial sources: BAF155 rabbit pAb (1:20; Santa Cruz), BAF155 mouse mAb (1:100; Santa Cruz), BAF170 rabbit pAb (1:100; Bethyl), BAF170 rabbit pAb (1:100; Sigma), Olig2 rabbit pAb (1:200; Millipore), PDGFRα rat pAb (1:200; BD Bioscience), Sox9 rabbit pAb (1:100; Millipore), Sox10 Guinea pig pAb (1:100; a gift from Prof. Michael Wegner), Ki67 rabbit pAb (1:50; Novocastra), Ki67 mouse mAb (1:100; Novocastra), IdU mouse mAb (1:50; Becton Dickinson), Casp3 rabbit pAb (1:100; Cell Signaling).

Secondary antibodies used were horseradish peroxidase (HRP)-conjugated goat anti-rabbit IgG (1:10,000; Covance), HRP-conjugated goat anti-mouse IgG (1:5,000; Covance), HRP-conjugated goat anti-rat IgG (1:10,000; Covance), and Alexa 488-, Alexa 568-, Alexa 594- and Alexa 647-conjugated IgG (various species, 1:400; Molecular Probes).

### RNA Sequencing

RNA was isolated from 4 control and 4 mutant dTel at P3 as previously described (Kiszka, 2019). Deep sequencing and data analysis were described previously (Narayanan et al., 2015; Nguyen et al., 2018). Briefly, cDNA libraries were prepared using the TruSeq RNA Sample Preparation v2 Kit. DNA was quantified using a NanoDrop spectrophotometer, and its quality was assessed using an Agilent 2100 Bioanalyzer. Reads were aligned to mouse genome mm10 and counted using FeaturesCount^1^. Differential expression was assessed using DESeq2 from Bioconductor (Love et al., 2014). Functional GO enrichment analyses were performed using ToppGene (Chen et al., 2009). The high-throughput RNA-seq data has be deposited in the NCBI Gene Expression Omnibus and made accessible through GEO Series accession number (GSE165228).

### *In situ* Hybridization

Chromogenic *in situ* hybridization (CISH) was performed in RNase-free condition as described previously (Tuoc et al., 2009). CISH was done on 10 μm sections from E12.5–E13.5 heads and on 16 μm sections from E15.5–E18.5 brains, which were fixed in 4% paraformaldehyde and cryoprotected according to Tuoc et al. (2009). The detection of the RNA transcripts of different RNA probes (riboprobes) was visualized via staining of the chromogen Digoxigenin (DIG)-marked specific riboprobes. RNA probes used in this study: *Olig1, Olig2, Sox10, and PLP*.

### Immunohistochemistry (IHC)

IHC was performed as previously described (Ulmke et al., 2021). Briefly, the antigen retrieval was performed by incubating the brain sections in 0.01 M sodium citrate buffer for 60 min at 70°C, followed by cool-down for 20 min at room temperature. The sections for IHC were then incubated overnight with primary antibody at 4°C after blocking with 5% normal sera of the appropriate species. Incubation with primary antibodies was followed by a 1 h incubation at room temperature with the appropriate A488-, A594-, A555- or A647-labeled (Alexa series, Invitrogen, 1:400) secondary goat or donkey antibodies. Sections were later counterstained with Vectashield mounting medium containing DAPI (Vector laboratories) to label nuclei.

### Imaging, Quantification, and Statistical Analyses

Images were captured using an Axio Imager M2 (Zeiss) with a Neurolucida system, and confocal fluorescence microscopes (TCS SP5; Leica). Images were further processed with Adobe Photoshop. IHC and ISH signal intensities were quantified by using Image J software, as previously described (Tuoc and Stoykova, 2008; Narayanan et al., 2015). For cell counts with the chromogenic signal, cells expressing the desired probes were counted directly by Neurolucida software within the desired area of the mouse forebrain. Statistical differences were measured using two-tailed unpaired Student’s *t*-test, with α set at 5% to give the following level of significance: ^∗^*p* ≤ 0.05, ^∗∗^*p* ≤ 0.01, ^∗∗∗^*p* ≤ 0.001, ^****^p ≤ 0.0001. All statistical graphs shown in this study were plotted by GraphPad Prism software (version 5). Adobe Illustrator CS6 was used to draw the schematic Figures: 2 (A) and 6. All details of statistical analyses for histological experiments are presented in Supplementary Table 2.

## Data Availability Statement

The data presented in the study are deposited in the GEO repository, accession number GSE165228.

## Ethics Statement

The animal study was reviewed and approved by Niedersächsisches Landesamt für Verbraucherschutz und Lebensmittelsicherheit, Georg-August-University Goettingen.

## Author Contributions

EA performed most characterization of dcKO phenotypes. EA, MH, and LP contributed to histological and data analyses. KK, CK, and AF generated RNA-sequencing data. JS and HN provided research tools and contributed to discussions. TT conceived and supervised the project. EA, MH, GS, and TT wrote the manuscript. All authors contributed to the article and approved the submitted version.

## Conflict of Interest

The authors declare that the research was conducted in the absence of any commercial or financial relationships that could be construed as a potential conflict of interest.

## Footnotes

1 http://bioinf.wehi.edu.au/featureCounts/

## References

[B1] Alver B. H. Kim K. H. Lu P. Wang X. Manchester H. E. Wang W. (2017). The SWI/SNF chromatin remodelling complex is required for maintenance of lineage specific enhancers. *Nat. Commun.* 8:14648. 10.1038/ncomms14648 28262751 PMC5343482

[B2] Bachmann C. Nguyen H. Rosenbusch J. Pham L. Rabe T. Patwa M. (2016). mSWI/SNF (BAF) Complexes Are Indispensable for the Neurogenesis and Development of Embryonic Olfactory Epithelium. *PLoS Genet.* 12:e1006274. 10.1371/journal.pgen.1006274 27611684 PMC5017785

[B3] Berry K. Wang J. Lu Q. R. (2020). Epigenetic regulation of oligodendrocyte myelination in developmental disorders and neurodegenerative diseases. *F1000Res.* 9 F1000FacultyRev–105. 10.12688/f1000research.20904.1 32089836 PMC7014579

[B4] Bischof M. Weider M. Küspert M. Nave K.-A. Wegner M. (2015). Brg1-Dependent Chromatin Remodelling Is Not Essentially Required during Oligodendroglial Differentiation. *J. Neurosci.* 35:21. 10.1523/JNEUROSCI.1468-14.2015 25568100 PMC6605249

[B5] Chen J. Bardes E. E. Aronow B. J. Jegga A. G. (2009). ToppGene Suite for gene list enrichment analysis and candidate gene prioritization. *Nucleic Acids Res.* 37 W305–W311. 10.1093/nar/gkp427 19465376 PMC2703978

[B6] Choi J. Ko M. Jeon S. Jeon Y. Park K. Lee C. (2012). The SWI/SNF-like BAF Complex Is Essential for Early B Cell Development. *J. Immunol.* 188 3791–3803. 10.4049/jimmunol.1103390 22427636

[B7] Copray S. Huynh J. Sher F. Casaccia P. Boddeke E. (2009). Epigenetic Mechanisms Facilitating Oligodendrocyte Development, Maturation, and Aging. *Glia* 57 1579–1587. 10.1002/glia.20881 19373939 PMC2760733

[B8] Dessaud E. Yang L. L. Hill K. Cox B. Ulloa F. Ribeiro A. (2007). Interpretation of the sonic hedgehog morphogen gradient by a temporal adaptation mechanism. *Nature* 450 717–720. 10.1038/nature06347 18046410

[B9] Egawa N. Shindo A. Hikawa R. Kinoshita H. Liang A. C. Itoh K. (2019). Differential roles of epigenetic regulators in the survival and differentiation of oligodendrocyte precursor cells. *Glia* 67 718–728. 10.1002/glia.23567 30793389 PMC6573028

[B10] Elsesser O. Fröb F. Küspert M. Tamm E. R. Fujii T. Fukunaga R. (2019). Chromatin remodeler Ep400 ensures oligodendrocyte survival and is required for myelination in the vertebrate central nervous system. *Nucleic Acids Res.* 47 6208–6224. 10.1093/nar/gkz376 31081019 PMC6614847

[B11] Emery B. (2010). Regulation of oligodendrocyte differentiation and myelination. *Science* 330 779–782. 10.1126/science.1190927 21051629

[B12] Emery B. Lu Q. R. (2015). Transcriptional and Epigenetic Regulation of Oligodendrocyte Development and Myelination in the Central Nervous System. *Cold Spring Harb. Perspect. Biol.* 7:a020461. 10.1101/cshperspect.a020461 26134004 PMC4563712

[B13] Finzsch M. Stolt C. C. Lommes P. Wegner M. (2008). Sox9 and Sox10 influence survival and migration of oligodendrocyte precursors in the spinal cord by regulating PDGF receptor alpha expression. *Development* 135 637–646. 10.1242/dev.010454 18184726

[B14] Galloway D. A. Moore C. S. (2016). miRNAs As Emerging Regulators of Oligodendrocyte Development and Differentiation. *Front. Cell Dev. Biol.* 4:59. 10.3389/fcell.2016.00059 27379236 PMC4911355

[B15] Gorski J. A. Talley T. Qiu M. Puelles L. Rubenstein J. L. Jones K. R. (2002). Cortical excitatory neurons and glia, but not GABAergic neurons, are produced in the Emx1-expressing lineage. *J. Neurosci.* 22 6309–6314. 10.1523/jneurosci.22-15-06309.2002 12151506 PMC6758181

[B16] Hargreaves D. C. Crabtree G. R. (2011). ATP-dependent chromatin remodeling: genetics, genomics and mechanisms. *Cell Res.* 21 396–420. 10.1038/cr.2011.32 21358755 PMC3110148

[B17] He D. Marie C. Zhao C. Kim B. Wang J. Deng Y. (2016). Chd7 cooperates with Sox10 and regulates the onset of CNS myelination and remyelination. *Nat. Neurosci.* 19 678–689. 10.1038/nn.4258 26928066 PMC4846514

[B18] Ho L. Crabtree G. R. (2010). Chromatin remodelling during development. *Nature* 463 474–484. 10.1038/nature08911 20110991 PMC3060774

[B19] Kessaris N. Fogarty M. Iannarelli P. Grist M. Wegner M. Richardson W. D. (2006). Competing waves of oligodendrocytes in the forebrain and postnatal elimination of an embryonic lineage. *Nat. Neurosci.* 9:173. 10.1038/nn1620 16388308 PMC6328015

[B20] Kiszka K. A. (2019). *A Guardian of Balance: The Role of BAF Chromatin Remodeling Complex in Astrogliogenesis During Mouse Forebrain Development. [dissertation]*. Goettingen: George-August-University Goettingen.

[B21] Koreman E. Sun X. Lu Q. R. (2018). Chromatin remodeling and epigenetic regulation of oligodendrocyte myelination and myelin repair. *Mol. Cell Neurosci.* 87 18–26. 10.1016/j.mcn.2017.11.010 29254827 PMC5828965

[B22] Lessard J. Wu J. I. Ranish J. A. Wan M. Winslow M. M. Staahl B. T. (2007). An essential switch in subunit composition of a chromatin remodeling complex during neural development. *Neuron* 55 201–215. 10.1016/j.neuron.2007.06.019 17640523 PMC2674110

[B23] Li H. He Y. Richardson W. D. Casaccia P. (2009). Two-tier transcriptional control of oligodendrocyte differentiation. *Curr. Opin. Neurobiol.* 19 479–485. 10.1016/j.conb.2009.08.004 19740649 PMC2826212

[B24] Li X. Liu G. Yang L. Li Z. Zhang Z. Xu Z. (2021). Decoding Cortical Glial Cell Development. *Neurosci. Bull.* 37 440–460. 10.1007/s12264-021-00640-9 33606177 PMC8055813

[B25] Limpert A. S. Bai S. Narayan M. Wu J. Yoon S. O. Carter B. D. (2013). NF-kappaB forms a complex with the chromatin remodeler BRG1 to regulate Schwann cell differentiation. *J. Neurosci.* 33 2388–2397. 10.1523/JNEUROSCI.3223-12.2013 23392668 PMC3711599

[B26] Love M. I. Huber W. Anders S. (2014). Moderated estimation of fold change and dispersion for RNA-seq data with DESeq2. *Genome Biol.* 15:550. 10.1186/s13059-014-0550-8 25516281 PMC4302049

[B27] Lu G. Zhang M. Wang J. Zhang K. Wu S. Zhao X. (2019). Epigenetic regulation of myelination in health and disease. *Eur. J. Neurosci.* 49 1371–1387. 10.1111/ejn.14337 30633380

[B28] Lu R. Barca O. (2012). Fine-Tuning Oligodendrocyte Development by microRNAs. *Front. Neurosci.* 6:13. 10.3389/fnins.2012.00013 22347159 PMC3272650

[B29] Maki T. Liang A. Miyamoto N. Lo E. Arai K. (2013). Mechanisms of oligodendrocyte regeneration from ventricular-subventricular zone-derived progenitor cells in white matter diseases. *Front. Cell. Neurosci.* 7:275. 10.3389/fncel.2013.00275 24421755 PMC3872787

[B30] Marathe H. G. Mehta G. Zhang X. Datar I. Mehrotra A. Yeung K. C. (2013). SWI/SNF enzymes promote SOX10- mediated activation of myelin gene expression. *PLoS One* 8:e69037. 10.1371/journal.pone.0069037 23874858 PMC3712992

[B31] Marie C. Clavairoly A. Frah M. Hmidan H. Yan J. Zhao C. (2018). Oligodendrocyte precursor survival and differentiation requires chromatin remodeling by Chd7 and Chd8. *Proc. Natl. Acad. Sci. U. S. A.* 115 E8246–E8255. 10.1073/pnas.1802620115 30108144 PMC6126750

[B32] Matsumoto S. Banine F. Feistel K. Foster S. Xing R. Struve J. (2016). Brg1 directly regulates Olig2 transcription and is required for oligodendrocyte progenitor cell specification. *Dev. Biol.* 413 173–187. 10.1016/j.ydbio.2016.04.003 27067865 PMC4851915

[B33] Matsumoto S. Banine F. Struve J. Xing R. B. Adams C. Liu Y. (2006). Brg1 is required for murine neural stem cell maintenance and gliogenesis. *Dev. Biol.* 289 372–383. 10.1016/j.ydbio.2005.10.044 16330018

[B34] Müller D. Cherukuri P. Henningfeld K. Poh C. H. Wittler L. Grote P. (2014). Dlk1 promotes a fast motor neuron biophysical signature required for peak force execution. *Science* 343 1264–1266. 10.1126/science.1246448 24626931

[B35] Narayanan R. Pham L. Kerimoglu C. Watanabe T. Castro Hernandez R. Sokpor G. (2018). Chromatin Remodeling BAF155 Subunit Regulates the Genesis of Basal Progenitors in Developing Cortex. *iScience* 4 109–126. 10.1016/j.isci.2018.05.014 30240734 PMC6147019

[B36] Narayanan R. Pirouz M. Kerimoglu C. Pham L. Wagener R. J. Kiszka K. A. (2015). Loss of BAF (mSWI/SNF) complexes causes global transcriptional and chromatin state changes in forebrain development. *Cell Rep.* 13 1842–1854. 10.1016/j.celrep.2015.10.046 26655900

[B37] Naruse M. Ishizaki Y. Ikenaka K. Tanaka A. Hitoshi S. (2017). Origin of oligodendrocytes in mammalian forebrains: a revised perspective. *J. Physiol. Sci.* 67 63–70. 10.1007/s12576-016-0479-7 27573166 PMC5368213

[B38] Nave K. A. Werner H. B. (2014). Myelination of the nervous system: mechanisms and functions. *Annu. Rev. Cell Dev. Biol.* 30 503–533. 10.1146/annurev-cellbio-100913-013101 25288117

[B39] Nguyen H. Kerimoglu C. Pirouz M. Pham L. Kiszka K. A. Sokpor G. (2018). Epigenetic regulation by BAF complexes limits neural stem cell proliferation by suppressing Wnt signaling in late embryonic development. *Stem Cell Rep.* 10 1734–1750. 10.1016/j.stemcr.2018.04.014 29779894 PMC5993560

[B40] Nguyen H. Sokpor G. Pham L. Rosenbusch J. Stoykova A. Staiger J. F. (2016). Epigenetic regulation by BAF (mSWI/SNF) chromatin remodeling complexes is indispensable for embryonic development. *Cell Cycle* 15 1317–1324. 10.1080/15384101.2016.1160984 26986003 PMC4889280

[B41] Ninkovic J. Steiner-Mezzadri A. Jawerka M. Akinci U. Masserdotti G. Petricca S. (2013). The BAF Complex Interacts with Pax6 in Adult Neural Progenitors to Establish a Neurogenic Cross-Regulatory Transcriptional Network. *Cell Stem Cell* 13 403–418. 10.1016/j.stem.2013.07.002 23933087 PMC4098720

[B42] Ohtomo R. Iwata A. Arai K. (2018). Molecular mechanisms of oligodendrocyte regeneration in white matter-related diseases. *Int. J. Mol. Sci.* 19:1743. 10.3390/ijms19061743 29895784 PMC6032201

[B43] Parras C. Marie C. Zhao C. Lu Q. R. (2020). Chromatin remodelers in oligodendroglia. *Glia* 68 1604–1618. 10.1002/glia.23837 32460418

[B44] Peru R. L. Mandrycky N. Nait-Oumesmar B. Lu Q. R. (2008). Paving the axonal highway: from stem cells to myelin repair. *Stem Cell Rev.* 4 304–318. 10.1007/s12015-008-9043-z 18759012

[B45] Richardson W. D. Kessaris N. Pringle N. (2006). Oligodendrocyte wars. *Nat. Rev. Neurosci.* 7 11–18. 10.1038/nrn1826 16371946 PMC6328010

[B46] Rowitch D. H. (2004). Glial specification in the vertebrate neural tube. *Nat. Rev. Neurosci.* 5 409–419. 10.1038/nrn1389 15100723

[B47] Rowitch D. H. Kriegstein A. R. (2010). Developmental genetics of vertebrate glial-cell specification. *Nature* 468 214–222. 10.1038/nature09611 21068830

[B48] Samudyata, Castelo-Branco G. Liu J. (2020). Epigenetic regulation of oligodendrocyte differentiation: from development to demyelinating disorders. *Glia* 68 1619–1630. 10.1002/glia.23820 32154951

[B49] Silbereis J. C. Nobuta H. Tsai H.-H. Heine Vivi M. McKinsey Gabriel L. Meijer Dimphna H. (2014). Olig1 Function Is Required to Repress Dlx1/2 and Interneuron Production in Mammalian Brain. *Neuron* 81 574–587. 10.1016/j.neuron.2013.11.024 24507192 PMC3979971

[B50] Sock E. Wegner M. (2019). Transcriptional control of myelination and remyelination. *Glia* 67 2153–2165. 10.1002/glia.23636 31038810

[B51] Sokpor G. Castro-Hernandez R. Rosenbusch J. Staiger J. F. Tuoc T. (2018). ATP-Dependent Chromatin Remodeling During Cortical Neurogenesis. *Front. Neurosci.* 12:226. 10.3389/fnins.2018.00226 29686607 PMC5900035

[B52] Sokpor G. Kerimoglu C. Nguyen H. Pham L. Rosenbusch J. Wagener R. (2021). Loss of BAF Complex in Developing Cortex Perturbs Radial Neuronal Migration in a WNT Signaling-Dependent Manner. *Front. Mol. Neurosci.* 14:687581. 10.3389/fnmol.2021.687581 34220450 PMC8243374

[B53] Sokpor G. Xie Y. Rosenbusch J. Tuoc T. (2017). Chromatin Remodeling BAF (SWI/SNF) Complexes in Neural Development and Disorders. *Front. Mol. Neurosci.* 10:243. 10.3389/fnmol.2017.00243 28824374 PMC5540894

[B54] Stolt C. C. Lommes P. Sock E. Chaboissier M. C. Schedl A. Wegner M. (2003). The Sox9 transcription factor determines glial fate choice in the developing spinal cord. *Genes Dev.* 17 1677–1689. 10.1101/gad.259003 12842915 PMC196138

[B55] Tiane A. Schepers M. Rombaut B. Hupperts R. Prickaerts J. Hellings N. (2019). From OPC to Oligodendrocyte: an Epigenetic Journey. *Cells* 8:1236. 10.3390/cells8101236 31614602 PMC6830107

[B56] Tuoc T. C. Boretius S. Sansom S. N. Pitulescu M. E. Frahm J. Livesey F. J. (2013). Chromatin Regulation by BAF170 Controls Cerebral Cortical Size and Thickness. *Dev. Cell* 25 256–269. 10.1016/j.devcel.2013.04.005 23643363

[B57] Tuoc T. C. Radyushkin K. Tonchev A. B. Pinon M. C. Ashery-Padan R. Molnar Z. (2009). Selective cortical layering abnormalities and behavioral deficits in cortex-specific Pax6 knock-out mice. *J. Neurosci.* 29 8335–8349. 10.1523/JNEUROSCI.5669-08.2009 19571125 PMC6665651

[B58] Tuoc T. C. Stoykova A. (2008). Trim11 modulates the function of neurogenic transcription factor Pax6 through ubiquitin-proteosome system. *Genes Dev.* 22 1972–1986. 10.1101/gad.471708 18628401 PMC2492742

[B59] Ulmke P. A. Sakib M. S. Ditte P. Sokpor G. Kerimoglu C. Pham L. (2021). Molecular Profiling Reveals Involvement of ESCO2 in Intermediate Progenitor Cell Maintenance in the Developing Mouse Cortex. *Stem Cell Rep.* 16 968–984. 10.1016/j.stemcr.2021.03.008 33798452 PMC8072132

[B60] Wegner M. (2001). Expression of transcription factors during oligodendroglial development. *Microsc. Res. Tech.* 52 746–752. 10.1002/jemt.1059 11276127

[B61] Wegner M. (2008). A matter of identity: transcriptional control in oligodendrocytes. *J. Mol. Neurosci.* 35 3–12. 10.1007/s12031-007-9008-8 18401762

[B62] Weider M. Küspert M. Bischof M. Vogl M. R. Hornig J. Loy K. (2012). Chromatin-remodeling factor Brg1 is required for Schwann cell differentiation and myelination. *Dev. Cell* 23 193–201. 10.1016/j.devcel.2012.05.017 22814607

[B63] Yu Y. Chen Y. Kim B. Wang H. Zhao C. He X. (2013). Olig2 targets chromatin remodelers to enhancers to initiate oligodendrocyte differentiation. *Cell* 152 248–261. 10.1016/j.cell.2012.12.006 23332759 PMC3553550

[B64] Zhao C. Dong C. Frah M. Deng Y. Marie C. Zhang F. (2018). Dual Requirement of CHD8 for Chromatin Landscape Establishment and Histone Methyltransferase Recruitment to Promote CNS Myelination and Repair. *Dev. Cell* 45 753–768.e8. 10.1016/j.devcel.2018.05.022 29920279 PMC6063525

[B65] Zhuo L. Theis M. Alvarez−Maya I. Brenner M. Willecke K. Messing A. (2001). hGFAP−cre transgenic mice for manipulation of glial and neuronal function in vivo. *Genesis* 31 85–94. 10.1002/gene.10008 11668683

