# Supplementary material for: Conditional Loss of BAF (mSWI/SNF) Scaffolding Subunits Affects Specification and Proliferation of Oligodendrocyte Precursors in Developing Mouse Forebrain
Source: Front Cell Dev Biol. 2021 Jul 15;9:619538. doi: 10.3389/fcell.2021.619538 (PMC8320002; doi:10.3389/fcell.2021.619538)
Supplement: Supplementary file 3 [file Data_Sheet_3.docx]

**Supplementary figures and tables**


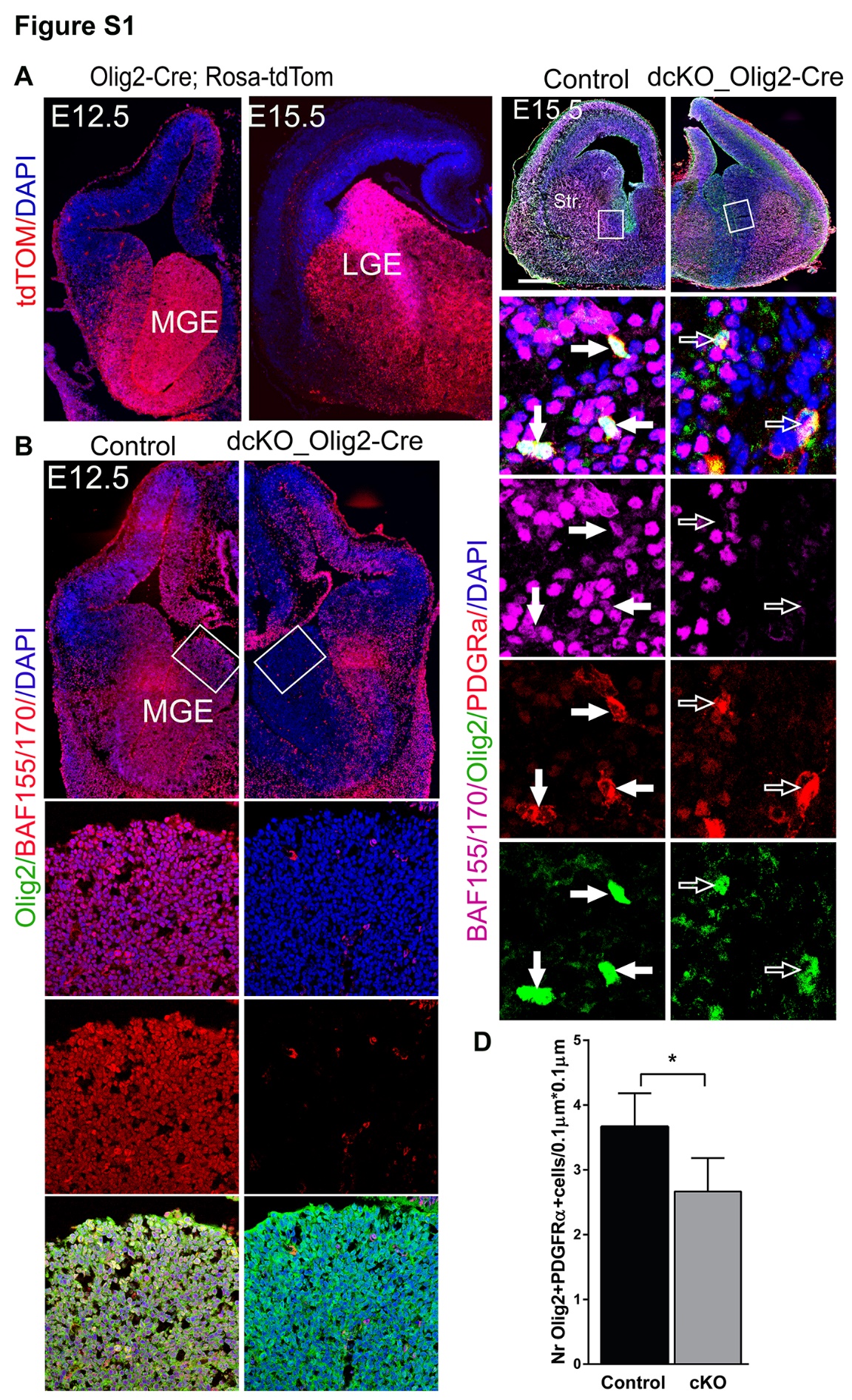


**Figure S1**. **Ablation of BAF155/BAF170 in mutant cortex is achieved early in corticogenesis and leads to loss of oligodendrocyte precursors.** (A) Immunomicrographs showing Olig2-Cre activity as Rosa-tdTomato in the medial ganglionic eminence (MGE) of the E12.5 mouse ventral telencephalon. By E15.5, the Olig2-Cre activity is extensive, covering the whole ventral telencephalon with prominence in the lateral ganglionic eminence (LGE). (B) Immunomicrographs showing massive loss of BAF155 and BAF170 in the MGE of the E12.5 mutant ventral telencephalon. Micrographs showing the E15.5 control and dcKO_Olig2-Cre forebrain immunostained with BAF177, BAF170, Olig2, and PDGRα antibodies. Images of higher magnification of regions, indicate with boxes in the overview images, in the control and mutant striatum (Str) are shown. Solid arrows point to cells in control striatum expressing BAF177, BAF170, Olig2, and PDGRα, whereas empty arrows indicate lack of BAF177 and BAF170 in such cells in the mutant striatum. (D) Bar graph showing reduced number of cells co-expression Olig2 and PDGRα in the E15.5 control striatum compared with that of dcKO_Olig2-Cre. Experimental replicates (n) = 6; *p ≤ 0.05. Scale bars = 100 μm.


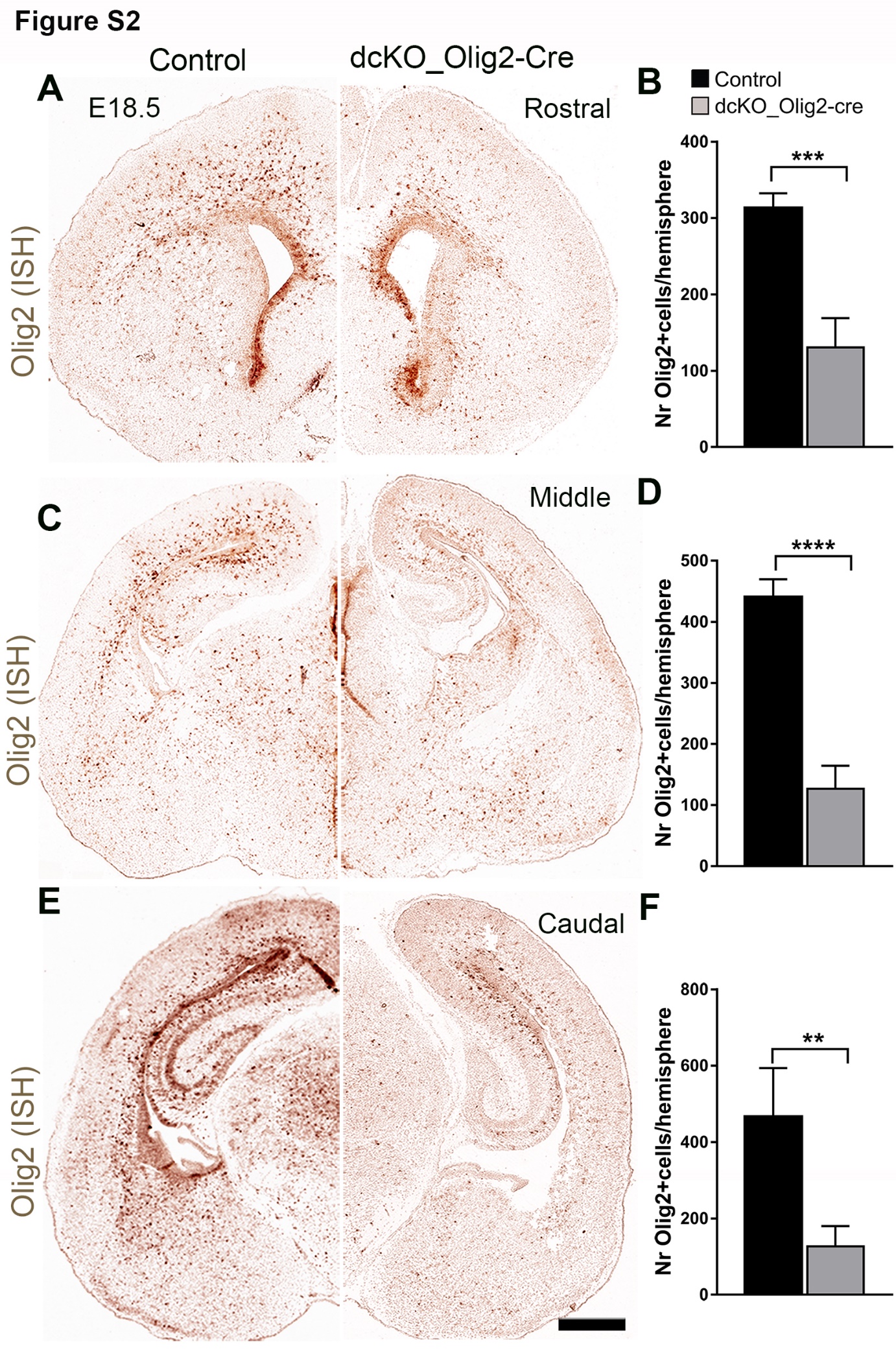


**Figure S2. Loss of Olig2-expressing cells in the perinatal mouse forebrain due to BAF155 and BAF170 ablation.** (A, C, E) *In situ* hybridization micrographs showing the E18.5 control and dcKO_Olig2-Cre rostrocaudal forebrain coronal sections riboprobed for Olig2 expression. (B, D, F) Bar graphs showing statistical quantification of Olig2-expressing cells in the E18.5 control and dcKO_Olig2-Cre at the rostral, middle, and caudal levels of the dorsal telencephalon. Data are shown as means ± SEMs. Experimental replicates (n) = 6; **p ≤ 0.01, ***p ≤ 0.001, ****p ≤ 0.0001. Scale bars = 100 μm.


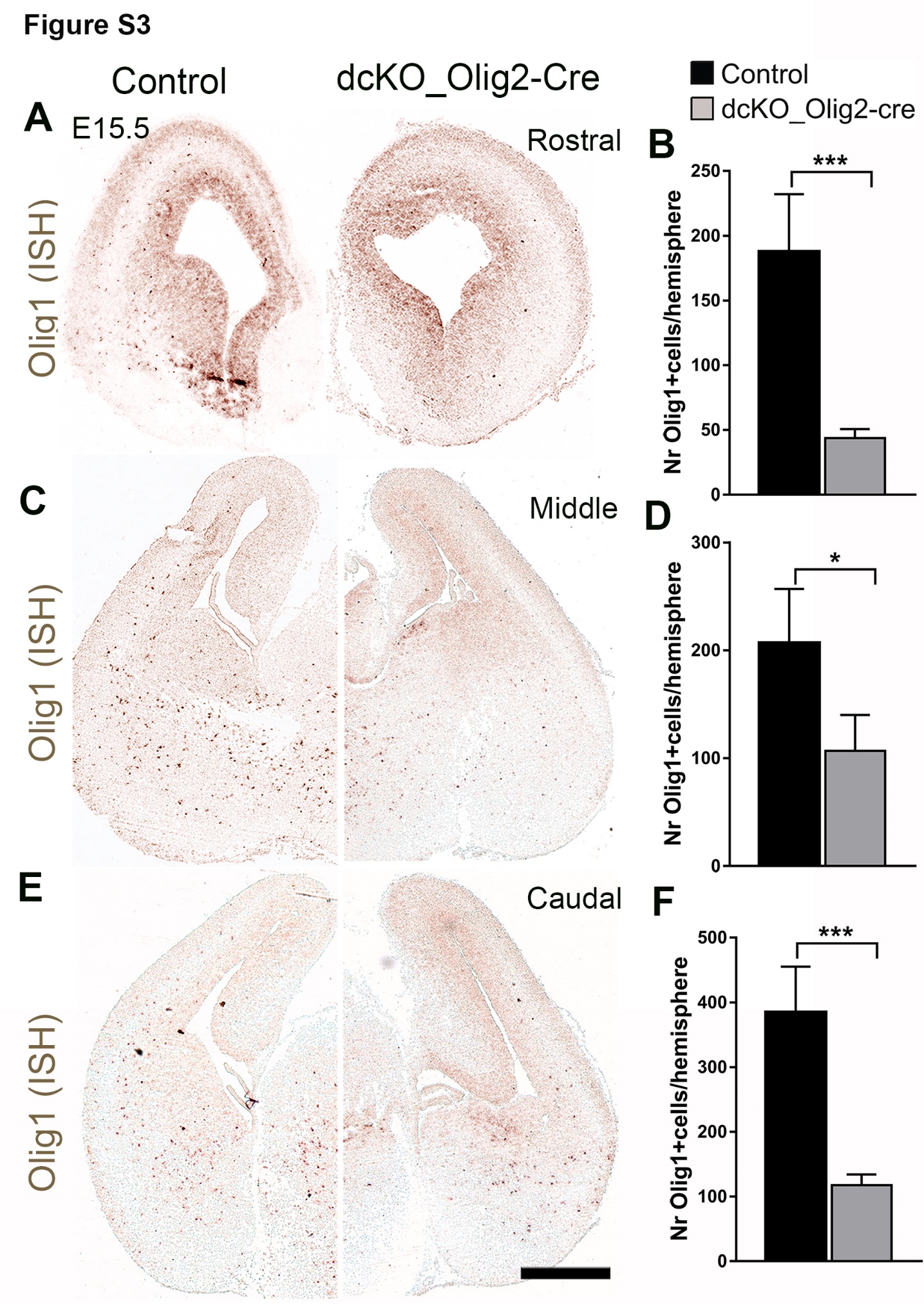


**Figure S3. BAF155 and BAF170-deficient forebrains display decreased numbers of Olig1-expressing cells.** (A, C, E) *In situ* hybridization micrographs showing the E15.5 control and dcKO_Olig2-Cre rostrocaudal forebrain coronal sections riboprobed for Olig1 expression. (B, D, F) Bar graphs showing statistical quantification of Olig1-expressing cells in the E15.5 control and dcKO_Olig2-Cre at the rostral, middle, and caudal levels of the dorsal telencephalon. Data are shown as means ± SEMs. Experimental replicates (n) = 6; *p ≤ 0.05, ***p ≤ 0.001. Scale bars = 100 μm.


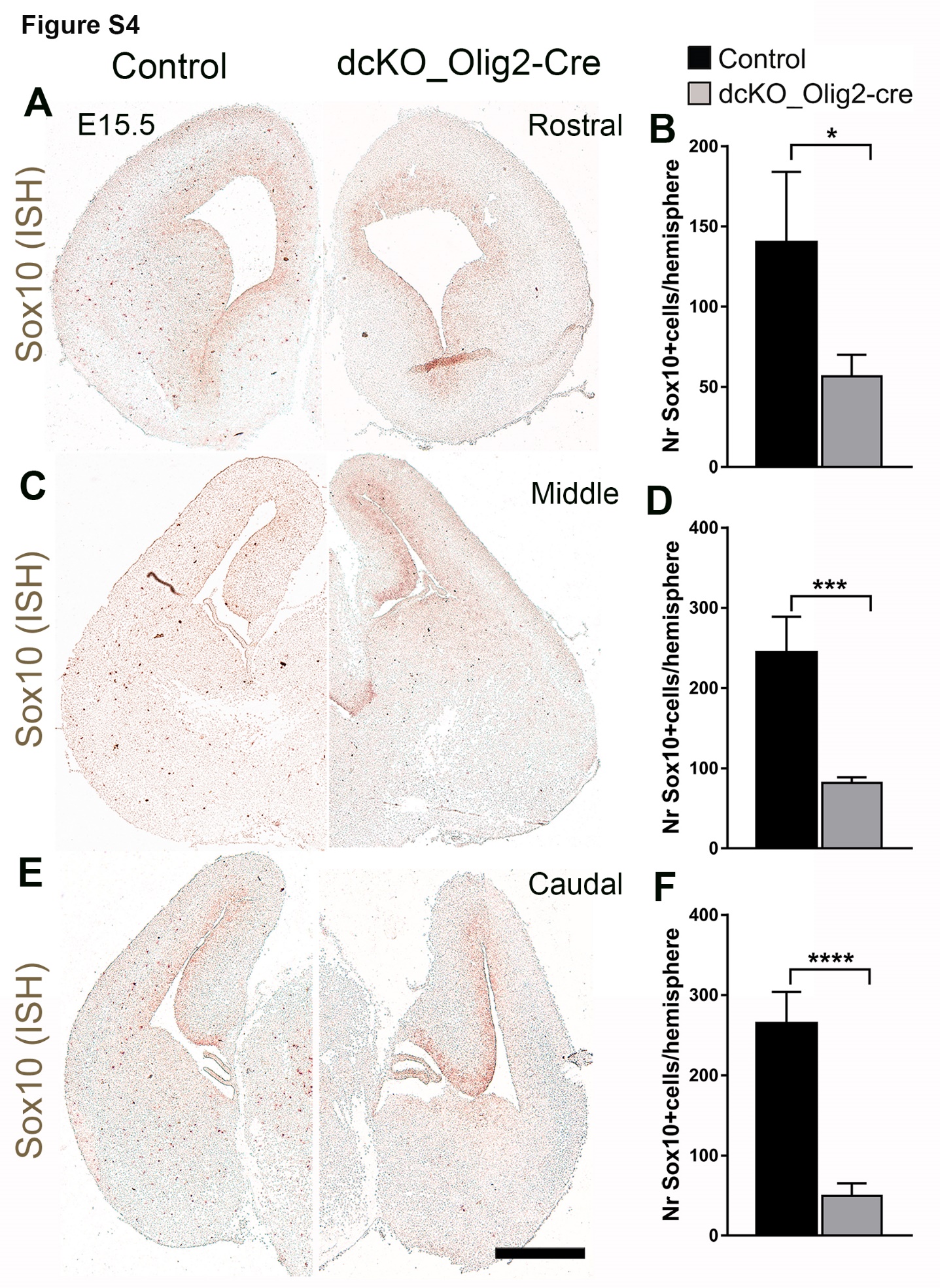


**Figure S4. Decreased number of Sox10-expressing cells due to loss of BAF155 and BAF170.** (A, C, E) *In situ* hybridization micrographs showing the E15.5 control and dcKO_Olig2-Cre rostrocaudal forebrain coronal sections riboprobed for Sox10 expression. (B, D, F) Bar graphs showing statistical quantification of Olig1-expressing cells in the E15.5 control and dcKO_Olig2-Cre at the rostral, middle, and caudal levels of the dorsal telencephalon. Data are shown as means ± SEMs. Experimental replicates (n) = 6; *p ≤ 0.05, ***p ≤ 0.001. Scale bars = 100 μm.


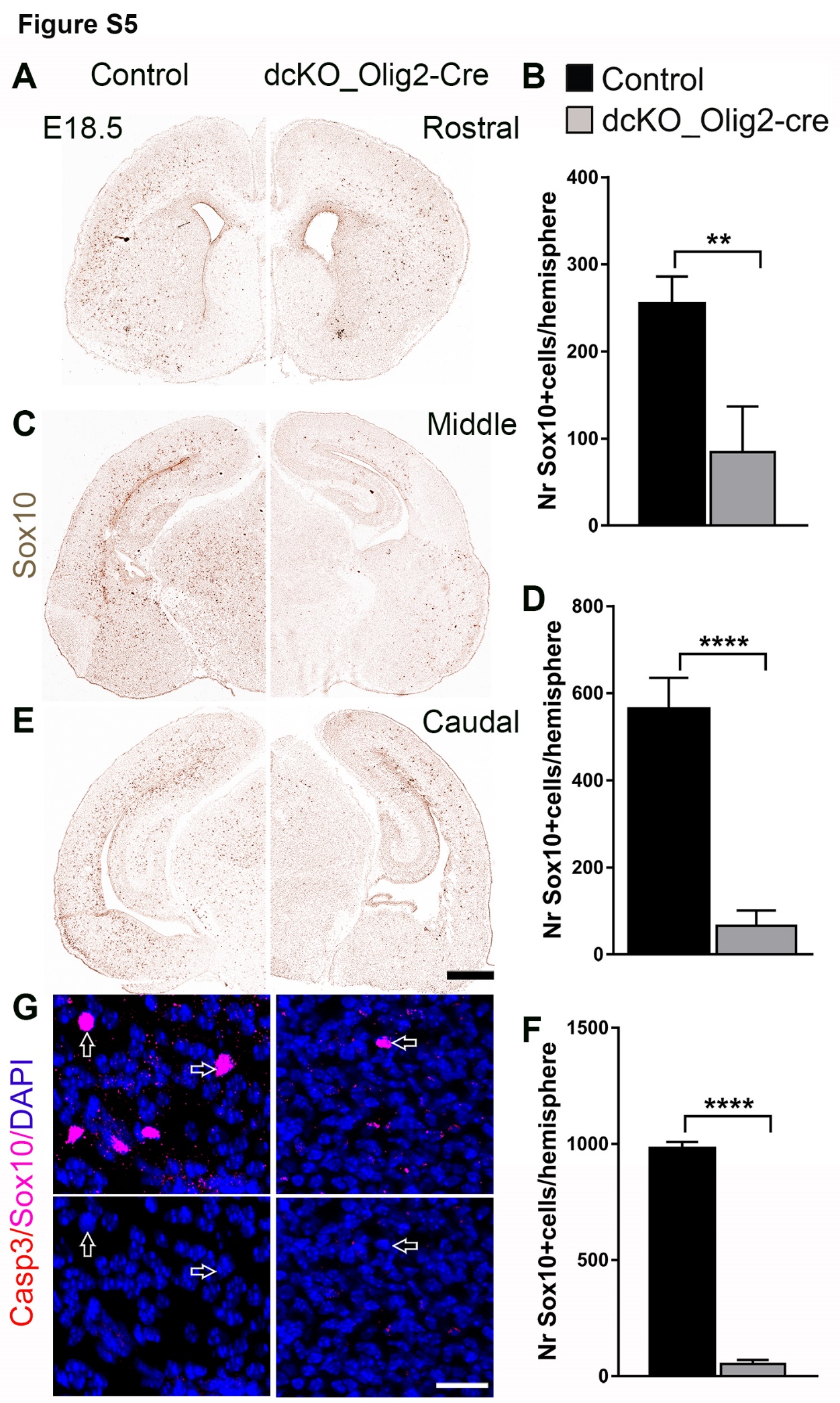


**Figure 5. BAF155 and BAF170 deletion in developing ventral telencephalon caused decrease in OPC differentiation.** (A, C, E) Micrographs showing *in situ* hybridization with the riboprobe Sox10, which marks immature differentiated OLs, in the control and dcKO_Olig2-Cre E18.5 mouse forebrain. (G) Immunomicrographs showing reduced Sox10 staining and comparable lack of Casp3 staining in the E18.5 mutant (dcKO_Olig2-Cre E18.5) and control mouse cortex. (B, D, F) Bar graphs showing quantification of the number of cells expressing Sox10+ along the rostrocaudal axis of the E18.5 control cortex compared with similar regions in the dcKO_Olig2_cre mutant. Inserted boxes in (A) show selected regions for quantifications. Values are expressed as means ± SEMs. Experimental replicates (n) = 6; **p ≤ 0.01, ****p ≤ 0.0001. Scale bars = 100 µm.


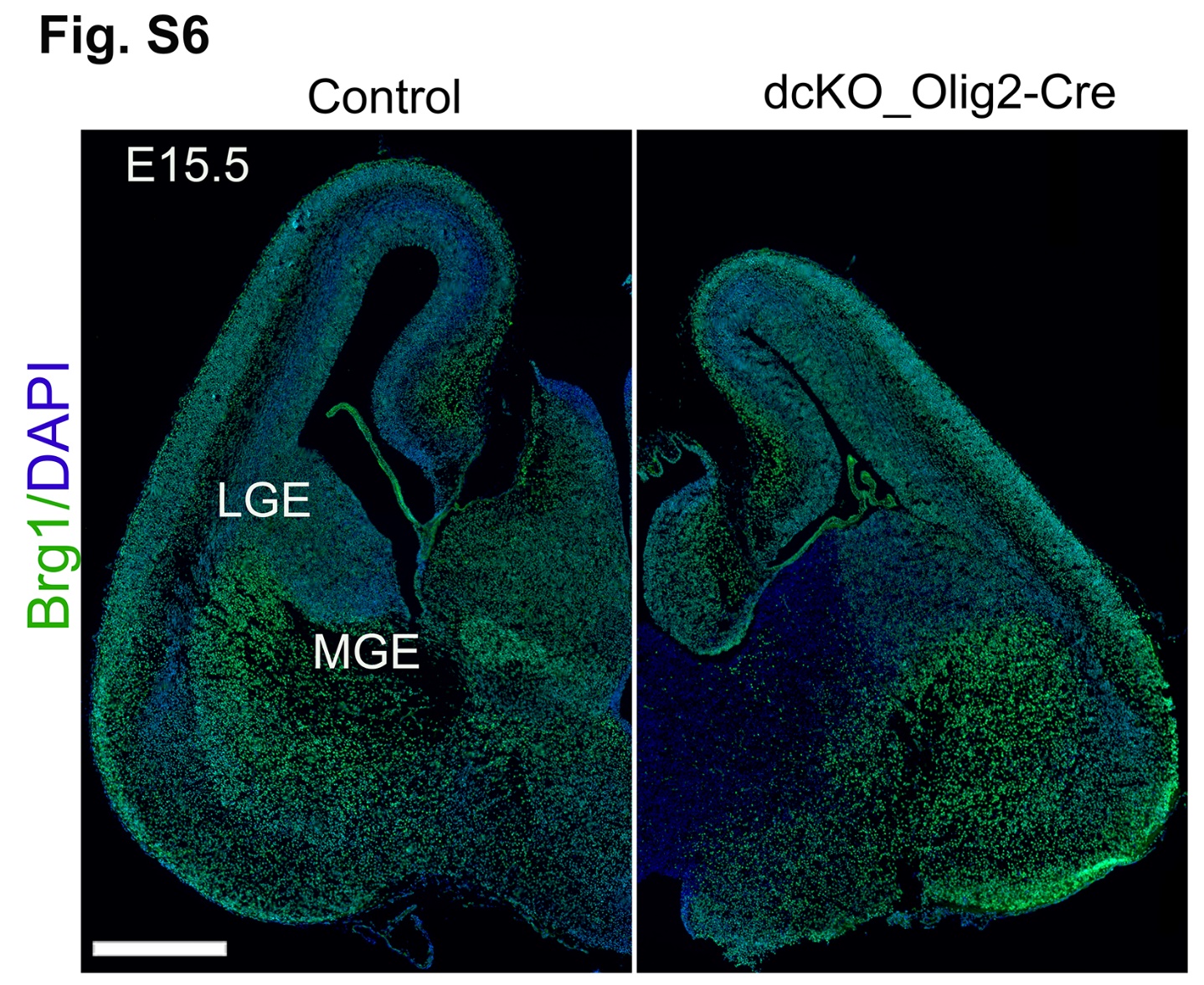


**Figure S6. Loss of Brg1 in medial ganglionic eminence due to deletion of BAF155 and BAF170 in striatum**. Immunomicrographs showing staining of Brg1 and DAPI counterstaining in the E15.5 control and dcKO_Olig2-Cre mouse forebrain.

**Table S1.** RNA-seq data in the dcKO_hGFAP-Cre cortex at P3 (as a separate datasheet)

**Table S2.** Statistical analysis (as a separate datasheet).
